# Atypical case of hantavirus infection in Sri Lanka mimicking leptospirosis: a case report

**DOI:** 10.1186/s13256-020-02417-6

**Published:** 2020-06-18

**Authors:** Chamara Dalugama, Madushi Nanayakkara, Nimanthi Rathnayaka, Arjuna Medagama

**Affiliations:** 1 Department of Medicine, University of Peradeniya, Peradeniya, Sri Lanka; 2 University Medical Unit, Teaching Hospital, Peradeniya, Sri Lanka

**Keywords:** Hantavirus, Hantavirus pulmonary syndrome, Hemorrhagic fever with renal syndrome, Leptospirosis, Multiorgan dysfunction

## Abstract

**Background:**

Hantavirus infection is an emerging zoonotic infection which has two characteristic patterns of presentation: hantavirus pulmonary syndrome and hemorrhagic fever with renal syndrome. The clinical presentation of hantavirus infection closely mimics leptospirosis.

**Case presentation:**

This case report describes a previously apparently well 36-year-old Sri Lankan Sinhalese man who presented with an acute febrile illness with myalgia, with liver involvement in the form of transaminitis, cardiac involvement in the form of myocarditis, acute kidney injury, and pulmonary involvement. He was initially managed as severe leptospirosis with multiorgan dysfunction with antibiotics, steroids, and N-acetyl cysteine. A diagnosis of acute hantavirus infection was made subsequently. He made an uneventful recovery.

**Conclusion:**

Hantavirus infections need to considered in the differential diagnosis of patients presenting with acute febrile illness with multiorgan involvement. Larger studies are needed to evaluate the seroprevalence of hantavirus in Sri Lanka because it could be an emerging serious public health problem.

## Introduction

Hantavirus infection is an emerging zoonotic disease caused by the virus hantavirus which is an ribonucleic acid (RNA) virus in the genus *Hantavirus* of the family *Bunyaviridae* [1]. Two characteristic disease patterns are described in hantavirus infections in humans: hantavirus pulmonary syndrome (HPS) and hemorrhagic fever with renal syndrome (HFRS) [1]. Although a significant number of cases of hantavirus are reported worldwide, cases reported in Sri Lanka are very few in number, probably because of low clinical suspicion and lack of diagnostic tests [2, 3].

Humans acquire the virus via a respiratory route by inhalation of aerosols contaminated with infected rodents’ feces, urine, or saliva [4], or, rarely, through direct contact with infected rodents’ faces or urine, or, rarely, from a bite from an infected rodent [5]. After reaching the lung parenchyma, the virus is taken up by phagocytes and migrated to regional lymph nodes; it is subsequently disseminated to distant organs including the heart, liver, and kidney. Involvement of the vascular endothelium of the heart, kidney, lung, and lymphoid organs with activation of both innate and acquired immune systems will lead to HPS and HFRS in susceptible individuals [6].

The initial clinical presentation includes fever with myalgia, conjunctival injection, icterus, hepatitis, myocarditis, and renal and lung involvement in the background of rodent exposure, which is similar to the presentation of leptospirosis [7, 8]. In the absence of widely available confirmatory tests, most cases of hantavirus are treated as leptospirosis.

We report a case of a previously well man with significant rodent exposure presenting clinically similar to leptospirosis with multiorgan involvement and subsequently diagnosed to have hantavirus infection.

## Case presentation

We report the case of a 36-year-old Sri Lankan Sinhalese man from Kandy, Sri Lanka, who presented to a tertiary care hospital with a 3-day history of an acute febrile illness. He had been in apparently good health and working as farmer involved in paddy cultivation. Three days prior to admission he developed high spiking fever with chills and rigors associated with severe arthralgia and myalgia. He could not mobilize due to severe muscle cramps in lower limbs. He developed shortness of breath at rest with a non-productive cough 1 day prior to admission and was anuric for 12 hours prior to hospital admission. His past medical history was unremarkable and there was no significant medical illness in his family. He was an occasional ethanol consumer and did not smoke tobacco.

On admission to our emergency unit, we found an averagely built man with a body mass index of 24 who was in severe distress and pain. He was severely dehydrated. He had mild icterus with injected and suffused conjunctiva. He had a temperature of 39.5 ºC with warm peripheries. His pulse rate was 140/minute with a blood pressure of 80/40 mmHg and he had marked postural symptoms on attempting a standing blood pressure. He was dyspneic with a respiratory rate of 32 cycles per minute on air saturation of 90%; it improved with 10 L oxygen via a face mask. On examination of his lung fields he had bilateral coarse crepitations. He had 3 cm hepatomegaly which was tender without palpable spleen or flank dullness. Although he was agitated and in distress, he was oriented in time, place, and person with normal neurology.

His laboratory results showed a leukocyte count of 24.6 × 10^9^/l (90% neutrophils) with a platelet count of 86 × 10^9^/l and hemoglobin of 14.5 g/dL. A peripheral smear showed neutrophil leukocytosis with toxic neutrophils, few myelocytes, and abnormal lymphocytes with thrombocytopenia. His aspartate aminotransferase (AST) level was 924 U/l (normal up to 31 U/l) and alanine aminotransferase (ALT) was 331 U/L (normal up to 31 U/L). His serum bilirubin level was 55 mmol/L (normal 1–21 mmol/L) with direct fraction of 55%. His alkaline phosphatase level was 459 U/L (normal 64–306 U/L). His serum creatinine was 217 micromoles/L on admission with a potassium of 2.9 mmol/L and sodium of 136 mmol/L. His blood urea nitrogen level was 40 mg/dl (normal 8–20 mg/dl). His coagulation profile was normal. His C-reactive protein (CRP) was 379 mg/dl on admission. Arterial blood gas revealed a partially compensated metabolic acidosis (pH 7.26) with an arterial bicarbonate of 14.5 mmol/L and carbon dioxide partial pressure of 20 mmHg. His creatinine kinase level was 440 mcg/l (10–120 mcg/l). His arterial lactate was 5 mmol/L. A chest X ray on admission showed bilateral air space opacifications. An electrocardiogram (ECG) showed T inversions in the anterior leads (V1–V6) with a troponin I of 0.16 ng/mL (0.04 ng/mL). Bedside two-dimensional echocardiography revealed global hypokinesia with a left ventricular ejection fraction of 40% which was suggestive of myocarditis.

A working diagnosis of severe leptospirosis with multiorgan dysfunction was made based on the above findings in the background of significant mud exposure and epidemiology of the locality. He was immediately moved to a high dependency unit and oxygen was given via a face mask. He had 50 ml of concentrated urine after catheterization. After fluid resuscitation of 1.5 L of crystalloids, he was started on intravenously administered noradrenalin as his blood pressure remained low. Intravenously administered cefotaxime with orally administered doxycycline was prescribed after taking blood cultures. Acidosis was corrected with 8.4% sodium bicarbonate. An N-acetyl cysteine (NAC) infusion was started considering his elevated transaminases. Hypokalemia was corrected with intravenously administered potassium chloride. He was given 1 g of intravenously administered methylprednisolone.

On day 2 of illness, our patient was clinically improved with less myalgia. Inotrope requirement was reduced from 0.8 mcg/kg per minute to 0.2 mcg/kg per minute. His oxygen requirement improved from 10 L/minute via face mask to 2 L/minute oxygen via nasal prongs. His urine output was 525 ml/last 24 hours.

On day 3 of illness his blood pressure was normalized without inotropes and on day 4 of illness his saturation on air was 99%.

Over the subsequent 5 days his clinical condition gradually improved with gradual normalization of biochemistry.

Methylprednisolone 1 g continued for 3 days and intravenously administered cefotaxime and orally administered doxycycline for a total of 7 days. He was discharged after an uneventful recovery on day 7 of hospital admission.

His dengue NS1 antigen test was negative on day 3 of fever. Throat and nasal swabs taken on admission were negative for influenza A (H1N1) viral RNA. A microscopic agglutination test (MAT) for IgM and IgG against *Leptospira* was done on day 7 and was negative. It was done in the Medical Research Institute of Sri Lanka, which is the reference center which tests for all the common serovars of leptospirosis in Sri Lanka. Blood taken on day 7 was positive for IgM against hantavirus on 1:100 dilutions. Unfortunately, our patient did not turn up for subsequent testing at 4 weeks to demonstrate the rising antibody titers in convalescence.

## Discussion

We report the case of a patient who presented from the farming community with possible rodent exposure with an acute febrile illness with severe myalgia and arthralgia. On admission, he had clinical features suggestive of cardiac, liver, and renal involvement with low blood pressure. Biochemistry confirmed the liver dysfunction with renal involvement. ECG and elevated troponin suggested possible myocardial involvement along with X-ray findings of bilateral pulmonary infiltrates which could be due to pneumonitis or pulmonary hemorrhages. These findings were very compatible with a severe leptospirosis infection with multiorgan dysfunction.

On this presentation we had few other possible alternative diagnoses. Dengue was considered a possibility as it was epidemiologically common and can present in a similar pattern in a complicated scenario with severe myocarditis, hepatitis, insufficiency, and myalgia [9, 10]. However, the elevated white blood cell count and very high inflammatory markers were against the diagnosis of dengue; subsequently, dengue was excluded by negative NS1 antigen and negative dengue IgM. Rickettsial infections can have a similar presentation with multiorgan involvement [11]. The possibility of atypical pneumonia was considered because our patient had cough with shortness of breath on admission. Hepatitis, myocarditis, and renal injury are well-known extrapulmonary manifestations of atypical pneumonia [12]. Severe influenza can also have a similar presentation with fever, myalgia, and type 1 respiratory failure with multiorgan dysfunction [13]. A diagnosis of hantavirus was considered due to the close resemblance of symptomatology with leptospirosis, although it is very infrequently reported [8].

Our patient was started on third-generation cephalosporin and doxycycline on admission which would cover the possibilities of leptospirosis, rickettsial infection, and atypical pneumonia. He was hydrated cautiously with intravenously administered fluid considering the possibility of myocarditis and started on inotropes with maintenance of mean arterial pressure of more than 90 mmHg.

The use of methylprednisolone was considered for our patient after carefully weighing the risks and benefits and after covering with broad-spectrum antibiotics. The use of methylprednisolone in severe leptospirosis was shown to be effective in some studies. Kularatne *et al.* have shown that methylprednisolone may reduce mortality in patients with severe leptospirosis except in cases with severe established organ failure [14]. Similar promising results were shown by Shenoy *et al*., that is, steroids reduce mortality when used early in the management of pulmonary leptospirosis [15]. Subsequently, our patient’s diagnosis was revisited with the positive hantavirus serology. The use of steroids in hantavirus was tested in a double-blind randomized case–control study which failed to demonstrate a significant clinical benefit to patients, although it appeared to be safe [16]. Although we need large randomized trials to evaluate the efficacy of steroids in severe hantavirus infection, we noticed a clear clinical and biochemical improvement following methylprednisolone administration.

Our patient had elevated transaminases on admission which was initially believed to be due to liver involvement in leptospirosis. However, subsequently, the diagnosis of hantavirus was entertained. Liver involvement in hantavirus is known. Elisaf *et al.* described a cohort of 32 patients with serologically confirmed hantavirus infection, 28% had elevated liver enzymes [17]. Elevated transaminases are described as an ominous prognostic factor. Icterus is not commonly reported, but hepatitis caused by hantavirus could lead to inflammation of hepatocytes and cause intrahepatic cholestasis. The use of NAC in patients with transaminitis is described in leptospirosis and dengue fever complicated with acute liver failure [9, 10, 18]. We started intravenously administered NAC in our patient and the infusion was continued until transaminases were less than 300. Our patient had a neutrophils leukocytosis with thrombocytopenia. The hematological abnormalities that were described in hantavirus infections include leukocytosis with increased myeloid elements and atypical lymphocytes and thrombocytopenia [19, 20].

Hantavirus infection in humans is described in two different clinical profiles. HFRS is described in a patient with serologically confirmed hantavirus with renal involvement in the form of hematuria, proteinuria, rising serum creatinine, and oliguria [21]. HPS leads to capillary leak into the pulmonary bed causing pulmonary edema, bronchorrhea, and myocarditis with cardiac arrhythmias [22]. Our patient had an overlap of both renal and cardiopulmonary involvement.

In Sri Lanka, hantavirus was first described in 1988 by Vitarana *et al.* by demonstrating positive serology in four patients among 248 tested who presented with leptospirosis-like illness [23]. In 2011, Gamage *et al*. demonstrated eight cases with previous exposure to hantavirus infection in Peradeniya hospital, Sri Lanka, during an outbreak of leptospirosis [24]. Recently, Ehelepola *et al*. described two atypical cases of hantavirus infection in Sri Lanka, where both had a combination of pulmonary and renal involvement similar to our patient [25]. Although infrequently described in Sri Lanka, we believe that hantavirus infection could be a significant emerging infection which is under reported owing to scarcity and non-availability of diagnostic facilities.

Serological tests are the mainstay of diagnosis of hantavirus infection, which include enzyme-linked immunosorbent assay (ELISA), strip immunoblot assay (SIA), Western blot, indirect immunofluorescence assay (IFA), complement fixation, hemagglutinin inhibition, and focus or plaque reduction neutralization tests to detect antibodies to hantaviruses. Reverse-transcription polymerase chain reaction (RT-PCR) can also be used to detect viral RNA [26]. We used IFA to detect immunoglobulin M (IgM) against hantavirus on 1:100 dilutions to confirm the diagnosis. IFA sensitivity for combined IgG and IgM analysis followed by repeat testing in 4 weeks for rising titer in the convalescence would be ideal to confirm the diagnosis. Unfortunately, our patient did not turn up in 4 weeks for serological testing. Ribavirin is a nucleoside analogue which has shown some promise in treatment of HFRS [27]. It was not used in our patient as the diagnosis was made retrospectively after apparent recovery and it is not widely available in state sector hospitals.

With the supportive treatment with intravenously administered fluids, inotropes, steroids, and NAC along with the antibiotics, our patient made a good recovery and was discharged without sequel on day 7 from admission.

## Conclusion

We believe that hantavirus should be considered in the differential diagnosis of a patient presenting with leptospirosis-like illness with multiorgan involvement. Larger community-based studies are needed to evaluate the seroprevalence of hantavirus infection among humans and domestic rodents because hantavirus could be a potentially emerging serious public health problem in Sri Lanka.

## Data Availability

Data sharing is not applicable to this article as no datasets were generated or analyzed during the current study.

## References

[1] Lee HW. Epidemiology and pathogenesis of hemorrhagic fever with renal syndrome. In: Elliott RM, editor. The Bunyaviridae. New York: Plenum Press; 1996. p. 253–67.

[2] Schmaljohn C, Hjelle B. Hantaviruses—a global disease problem. Emerg Infect Dis. 1997;3:95–104. 10.3201/eid0302.970202 PMC2627612 9204290

[3] Agampodi S, Peacock SJ, Thevanesam V. The potential emergence of leptospirosis in Sri Lanka. Lancet Infect Dis. 2009;9(9):524–6. 10.1016/S1473-3099(09)70211-7. 19695489

[4] Mertz GJ, Hjelle BL, Bryan RT. Hantavirus infection. Adv Intern Med. 1997;42:369. 9048125

[5] Centers for Disease Control and Prevention. Hantavirus. 2019. www.cdc.gov/hantavirus.

[6] Safronetz D, Prescott J, Feldmann F, *et al.* Pathophysiology of hantavirus pulmonary syndrome in rhesus macaques. Proc Natl Acad Sci U S A. 2014;111(19):7114–9. 10.1073/pnas.1401998111. PMC4024883 24778254

[7] Davies EA, Rooney PJ, Coyle PV, Simpson DI, Montgomery IW, Stanford CF. Hantavirus and Leptospira. Lancet. 1998;2:460–1. 10.1016/s0140-6736(88)90458-8 2900396

[8] Dahanayaka NJ, Agampodi SB, Bandaranayaka AK, Priyankara S, Vinetz JM. Hantavirus infection mimicking leptospirosis: how long are we going to rely on clinical suspicion? J Infect Dev Ctries. 2014;8(8):1072–5. 10.3855/jidc.4115. 25116678

[9] Dalugama C, Gawarammana IB. Lessons learnt from managing a case of dengue hemorrhagic fever complicated with acute liver failure and acute kidney injury: a case report. J Med Case Rep. 2018;12(1):215. 10.1186/s13256-018-1766-0. Published 2018 Aug 8. PMC6081819 30089506

[10] Dalugama C, Gawarammana IB. Dengue hemorrhagic fever complicated with acute liver failure: a case report. J Med Case Rep. 2017;11(1):341. 10.1186/s13256-017-1510-1. Published 2017 Dec 8. PMC5721681 29216924

[11] Kularatne SA, Edirisingha JS, Gawarammana IB, Urakami H, Chenchittikul M, Kaiho I. Emerging rickettsial infections in Sri Lanka: the pattern in the hilly Central Province. Tropical Med Int Health. 2003;8(9):803–11. 10.1046/j.1365-3156.2003.01108.x 12950666

[12] Zhai SB, Cao DB, Hui-Xu, Li XX, Yang SR. Multiple organ dysfunction syndrome associated with Mycoplasma pneumoniae infection. Braz J Microbiol. 2012;43(1):230–4. 10.1590/S1517-838220120001000025. PMC3768962 24031822

[13] Duran C, Taskar V. Influenza A precipitating multiorgan failure syndrome in sickle cell disease. Chest. 2018;154(4):284A.

[14] Kularatne SA, Budagoda BD, de Alwis VK, Wickramasinghe WM, Bandara JM, Pathirage LP, Gamlath GR, Wijethunga TJ, Jayalath WA, Jayasinghe C, Pinto V, Somaratne P, Kumarasiri PV. High efficacy of bolus methylprednisolone in severe leptospirosis: a descriptive study in Sri Lanka. Postgrad Med J. 2011;87(1023):13–7. 10.1136/pgmj.2009.092734. 21106802

[15] Shenoy VV, Nagar VS, Chowdhury AA, Bhalgat PS, Juvale NI. Pulmonary leptospirosis: an excellent response to bolus methylprednisolone. Postgrad Med J. 2006;82(971):602–6. 10.1136/pgmj.2005.044255. PMC2585719 16954459

[16] Vial PA, Valdivieso F, Ferres M, *et al.* High-dose intravenous methylprednisolone for hantavirus cardiopulmonary syndrome in Chile: a double-blind, randomized controlled clinical trial. Clin Infect Dis. 2013;57(7):943–51. 10.1093/cid/cit394. PMC3765009 23784924

[17] Elisaf M, Stefanaki S, Repanti M, Korakis H, Tsianos E, Siamopoulos KC. Liver involvement in hemorrhagic fever with renal syndrome. J Clin Gastroenterol. 1993;17:33–7.. 10.1097/00004836-199307000-00010 8104972

[18] Wysocki J, Liu Y, Shores N. Leptospirosis with acute liver injury. Proc (Bayl Univ Med Cent). 2014;27(3):257–8. 10.1080/08998280.2014.11929130. PMC4059585 24982581

[19] Gajdusek DC. Virus hemorrhagic fevers: special reference to hemorrhagic fever with renal syndrome (epidemic hemorrhagic fever). J Pediatr. 1962;60:841–57. 10.1016/s0022-3476(62)80170-x 13896278

[20] Lee HW. Hemorrhagic fever with renal syndrome in Korea. Rev Infect Dis. 1989;11(Suppl 4):S864–76. 2568676

[21] Niklasson B, Le Duc J. Isolation of the nephropathia epidemica agent in Sweden. Lancet. 1984;1:1012–3.. 10.1016/s0140-6736(84)92344-4 6143925

[22] Jenison S, Hjelle B, Simpson S, *et al.* Hantavirus pulmonary syndrome: clinical, diagnostic, and virologic aspects. Semin Respir Infect. 1995;10:259. 8668854

[23] Vitarana T, Colombage G, Bandaranayake V, Lee HW. Hantavirus disease in Sri Lanka. Lancet. 1988;2:1263.. 2904003

[24] Gamage CD, Yasuda SP, Nishio S, Kularatne SA, Weerakoon K, Rajapakse J, Nwafor-Okoli C, Lee RB, Obayashi Y, Yoshimatsu K, Arikawa J, Tamashiro H. Serological evidence of Thailand virus-related hantavirus infection among suspected leptospirosis patients in Kandy, Sri Lanka. Jpn J Infect Dis. 2011;64:72–5. 21266762

[25] Ehelepola NDB, Basnayake BMLS, Sathkumara SMBY, Kaluphana KLR. Two atypical cases of Hantavirus infections from Sri Lanka. Case Rep Infect Dis. 2018;2018:4069862. 6 pages. 10.1155/2018/4069862 PMC5933029 29850303

[26] Hjelle B, Jenison S, Torrez-Martinez N, *et al.* Rapid and specific detection of Sin Nombre virus antibodies in patients with hantavirus pulmonary syndrome by a strip immunoblot assay suitable for field diagnosis. J Clin Microbiol. 1997;35:600. 10.1128/jcm.35.3.600-608.1997 PMC229635 9041397

[27] Huggins JW, Hsiang CM, Cosgriff TM, *et al.* Prospective, double-blind, concurrent, placebo-controlled clinical trial of intravenous ribavirin therapy of hemorrhagic fever with renal syndrome. J Infect Dis. 1991;164:1119. 10.1093/infdis/164.6.1119 1683355

